# HMPDACC: a Human Microbiome Project Multi-omic data resource

**DOI:** 10.1093/nar/gkaa996

**Published:** 2020-12-10

**Authors:** Heather Huot Creasy, Victor Felix, Jain Aluvathingal, Jonathan Crabtree, Olukemi Ifeonu, James Matsumura, Carrie McCracken, Lance Nickel, Joshua Orvis, Mike Schor, Michelle Giglio, Anup Mahurkar, Owen White

**Affiliations:** Institute for Genome Sciences, University of Maryland School of Medicine, Baltimore, MD 21201, USA; Institute for Genome Sciences, University of Maryland School of Medicine, Baltimore, MD 21201, USA; Institute for Genome Sciences, University of Maryland School of Medicine, Baltimore, MD 21201, USA; Institute for Genome Sciences, University of Maryland School of Medicine, Baltimore, MD 21201, USA; Institute for Genome Sciences, University of Maryland School of Medicine, Baltimore, MD 21201, USA; Institute for Genome Sciences, University of Maryland School of Medicine, Baltimore, MD 21201, USA; Institute for Genome Sciences, University of Maryland School of Medicine, Baltimore, MD 21201, USA; Institute for Genome Sciences, University of Maryland School of Medicine, Baltimore, MD 21201, USA; Institute for Genome Sciences, University of Maryland School of Medicine, Baltimore, MD 21201, USA; Institute for Genome Sciences, University of Maryland School of Medicine, Baltimore, MD 21201, USA; Institute for Genome Sciences, University of Maryland School of Medicine, Baltimore, MD 21201, USA; Institute for Genome Sciences, University of Maryland School of Medicine, Baltimore, MD 21201, USA; Institute for Genome Sciences, University of Maryland School of Medicine, Baltimore, MD 21201, USA

## Abstract

The Human Microbiome Project (HMP) explored microbial communities of the human body in both healthy and disease states. Two phases of the HMP (HMP and iHMP) together generated >48TB of data (public and controlled access) from multiple, varied omics studies of both the microbiome and associated hosts. The Human Microbiome Project Data Coordination Center (HMPDACC) was established to provide a portal to access data and resources produced by the HMP. The HMPDACC provides a unified data repository, multi-faceted search functionality, analysis pipelines and standardized protocols to facilitate community use of HMP data. Recent efforts have been put toward making HMP data more findable, accessible, interoperable and reusable. HMPDACC resources are freely available at www.hmpdacc.org.

## INTRODUCTION

Any study of human health and disease must take into consideration the multitudes of microorganisms that call the human body home. In order to gain a complete understanding of the human host/microbiota holobiont, the delicate interactions between human physiology and microbial physiology in the environment of the human body must be deciphered. Since the majority of microbes residing in the human body remain uncultured, and thus uncharacterized in a traditional laboratory setting, the tools of 16S amplicon, metagenome and metatranscriptome sequencing are used to study these populations of mixed species.

The NIH Common Fund initiated the Human Microbiome Project (HMP) (http://hmpdacc.org/) in 2008 to explore the microbial communities of the human host and characterize their role in human health and disease. The overall goals of the initial 5-year phase of the HMP effort were to establish a baseline of data from a large sample of healthy subjects, explore possible correlations between changes in community compositions and disease states, and to provide resources for the research community to use in human microbiome research and analysis. Over 11 000 physical samples were collected from 300 healthy subjects, from which nearly 10 000 were selected for analysis. To constrain cost, all selected samples underwent 16S sequencing using a variety of primer sets, while a strategically selected subset of ∼2300 samples underwent metagenomic whole shotgun sequencing (wgs). Primary sequence data were generated and used for secondary analyses that included community profiling, metagenome assembly, gene prediction, functional annotation, pathway profiling and gene clustering. Furthermore, the project produced numerous additional resources including isolate reference genome sequences, software tools, protocols and web resources (1).

A 3-year second phase of the HMP, the Integrative Human Microbiome Project (iHMP), involved studies focusing on three particular conditions in human health (2,3): onset of Type 2 diabetes (4), inflammatory bowel disease (5) and pregnancy/pre-term birth (6). In contrast to the initial phase of the HMP project, many different types of omics approaches were carried out on both the microbiome and the host in order to provide a more comprehensive view of human–microbe interactions. Data generated for this effort included not only 16S and metagenomic wgs, but also transcriptome, proteome and metabolome investigations for both the host and microbiome, as well as host whole genomes and epigenomes.

Collection and organization of the large amounts of data and resources generated across both phases of the project is essential in order for the data to be used by the research community. To meet this need, a dedicated data coordination center was established here at the Institute for Genome Sciences, University of Maryland School of Medicine. For the first phase of the HMP, this resource was referred to as the HMP Data Analysis and Coordination Center (HMP-DACC). During the iHMP, this resource was referred to as the iHMP Data Coordination Center (iHMP-DCC). We will henceforth refer to both phases of data coordination resources as the HMPDACC.

Biological findings and overarching perspectives of the HMP and iHMP have been extensively described elsewhere (1,7,8) (https://collections.plos.org/hmp). Here we focus specifically on our work as the HMPDACC to coordinate and disseminate data and analyses resulting from the HMP and iHMP through our unified HMP/iHMP Data Coordination Center web resources. HMPDACC web resources were launched in 2009, initially providing information on all aspects of the HMP Phase 1 effort including: project information, primary sequence data, derived analysis results, software tools, protocols and step-by-step pipeline walkthroughs. A second site was developed for the iHMP DCC in early 2015. In 2017, we completed a merger of the two sites under the www.hmpdacc.org url. The merged site has experienced ∼120 000 user sessions each year for the last 4 years. While the overview and informational sections of the site remain separated by phase, based on historical and NIH Common Fund-driven separations, data associated with either HMP phase can be accessed through a single HMPDACC Data Portal. The result of HMPDACC efforts has made the HMP data more findable, accessible, interoperable and reusable (FAIR).

## RESOURCE CONTENT

### Sequence and analysis data

The HMPDACC Data Portal contains 48TB of data, associated with over 31 000 samples and 20 data types ranging from raw to processed from both host and microbiome (Table 1). Data were generated from different cohorts arising from various aspects of the overall HMP effort. Raw and derived data are available through the HMPDACC Data Portal, with raw, non-human data also available from NCBI Sequence Read Archive (SRA) (BioProject PRJNA43021, PRJNA395569, PRJNA430481, PRJNA497499). BMtagger (ftp://ftp.ncbi.nlm.nih.gov/pub/agarwala/bmtagger/) was used to screen out human sequences from metagenomic wgs data. Restricted-access human sequence data and protected metadata are available via dbGap, accessible through the above NCBI BioProject ids. Data can be grouped into the following categories:

**Table 1.** Data types, file counts and compressed file sizes of respective types currently available through the HMPDACC Data Portal. The first phase of the HMP focused on healthy human subjects and demonstration projects, as described in the text. This phase generated two datatypes: 16S and metagenomic wgs sequence. The second phase, the iHMP, was an integrated multi-omic analysis across three phenotypic projects. This phase generated a variety of multi-omic data types from both the host and the microbiome. Data types not generated by a phase or study have been grayed out. Across the right-most and bottom totals are the full complement of publicly available data within the HMPDACC Data Portal. An asterisk indicates controlled-access data types, which are not available through the HMPDACC Data Portal, accessible only to authorized users via dbGap

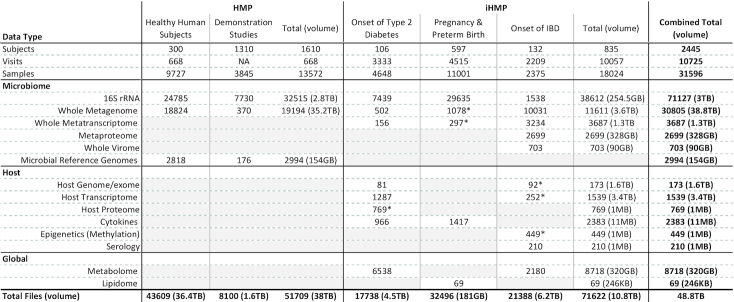

#### HMP phase one healthy human subjects (HHS)

The healthy human subjects study involved sampling 300 healthy individuals from five major body sites (airways, skin, oral cavity, gastrointestinal tract and vagina) at up to three timepoints (9). Each body site, except gut, was sampled at numerous subsites, for a total of up to 15 (male) or 18 (female) samples per individual per time point, resulting in over 11 000 samples. Metagenomic wgs data are available for 2355 samples and 16S amplicon sequence data for 9727 samples, with a subset of samples having undergone both metagenomic wgs and 16S sequencing. 16S community profiling (performed either by the HMPDACC or HMP Research Network Consortium members) included QIIME (10) analysis and, for a subset of samples, mothur (11) analysis. For all HHS cohort metagenomic wgs samples, we provide community and functional profiling, initially by mapping to reference genomes, and subsequently using Metaphlan 2 and HUMAnN2 (12–14). HHS samples were assembled using IDBA-UD (15), and annotated using metagenemark 3.25 (16) and Attributor (https://github.com/jorvis/Attributor) to generate gene indices. Attributor examines a ranked list of sequence-based search results and uses the highest ranking, i.e. highest quality and most informative, piece of evidence associated with each predicted protein to make functional annotations. In addition to generating single-sample-based metagenomic wgs assemblies, we also evaluated the additional value of building co-assemblies using samples from the same subject and body site across multiple visits (8). Initial phylogenetic and functional analyses of the HHS cohort were published in 2012 (1,7) (https://collections.plos.org/hmp), with follow-up analyses, which included subsequently sequenced samples, published in 2017 (8). Raw and analysis data from all of the above activities are available through HMPDACC resources.

#### HMP phase one ‘Demonstration Studies’

Fifteen studies were funded as part of HMP phase one in order to examine the relationship between microbiome and disease. These studies addressed a wide range of health conditions involving several different body sites. Each involved one or more human cohorts including controls and affected individuals, and produced either metagenomic wgs or 16S sequence data, or in some cases, both. Raw data are available through the HMPDACC Data Portal.

#### HMP phase two: iHMP

The second phase of the HMP, the integrative HMP (iHMP) (2,3), expanded on the demonstration studies from phase one by focusing on three specific health areas—diabetes, inflammatory bowel disease and pregnancy. These studies involved a deeper investigation of the relationship between the microbiome and disease by generating and integrating a much wider array of omics data including, but not limited to, host genomes, host exomes, host transcriptomes, metagenomes, metatranscriptomes, proteomes and metabolomes (Table 1). In addition, abundant clinical metadata was captured. Raw and analysis data from the iHMP are available through the HMPDACC Portal.

#### Reference genomes

During the first phase of the HMP, a total of 2802 isolate reference genomes with demonstrated associations with the healthy human body were sequenced (17) to varying degrees of completeness and submitted to NCBI under BioProject 28331 (https://www.ncbi.nlm.nih.gov/bioproject/28331). The HMPDACC processed all HMP reference genomes through the GALES (https://github.com/jorvis/GALES) annotation pipeline that generated structural annotation from Prodigal (18) and functional annotation from Attributor (https://github.com/jorvis/Attributor). The HMPDACC provides assembled genome sequences as well as structural and functional annotations. Reference genome metadata is available in the HMPDACC Project Catalog, https://www.hmpdacc.org/hmp/catalog/.

### Metadata

All metadata associated with the HMP HHS cohort was submitted to dbGaP (dbGaP accession phs000228) and is available to authorized researchers. The HMP Steering Committee and associated IRBs approved public release of a limited subset of metadata fields, specifically patient and sample identifiers, gender, sample body site and visit number, available, where applicable, as faceted search objects on the HMPDACC Data Portal, or from the metadata project catalog of the HMPDACC website. The demonstration study and iHMP project cohorts vary in terms of publicly available metadata fields depending on institutional IRB approvals, with protected metadata again available at dbGaP. Sample, 16S and metagenomic wgs sequence-related metadata stored in the HMPDACC data resources are curated according to the GSC standards for Minimum Information about a Marker Gene Sequence (MIMARKS) or Minimum Information about a Metagenomic Sequence (MIMS), respectively (19) and are available as additional filters of the HMPDACC Data Portal faceted search.

### Tools and protocols

Several tools were used and/or specifically developed for analysis of HMP data. Standardized protocols were developed by, and used across all of, the sequencing and analysis centers associated with the phase one HMP HHS cohort analysis. These protocols formed the basis for updated strategies for follow-up analysis of HMP samples from the healthy subjects. As standardized protocols are not always consistently effective across different body sites, the demonstration projects and iHMP projects developed their own individual strategies and protocols, using the lessons learned during the initial rounds of HMP protocol development.

The HMPDACC website contains an area dedicated to protocols and tools used by the HMP Consortium (https://www.hmpdacc.org/hmp/resources/), allowing users to replicate analyses on HMP or custom datasets. In addition to pipelines used for previously published analyses, this section includes two pipelines recently developed by the HMPDACC, incorporating state-of-the-art tools, for reanalysis of HMP data or analysis of newly produced data. These pipelines are built within Docker containers, allowing them to be easily downloaded and run locally or in cloud environments. For processing of whole metagenome sequence data, we have developed a pipeline that includes KneadData (https://bitbucket.org/biobakery/kneaddata) and HUMAnN2 (14). This pipeline produces quality checked read files as well as community and metabolic profiling analysis results. For processing of 16S amplicon sequence data, we are in the final stages of developing and testing a pipeline that uses DADA2 (20). Both pipelines are freely available through dockerhub (https://hub.docker.com/u/umigs) or from the Resources tab of the HMPDACC website.

## DATA STORAGE INFRASTRUCTURE

### Data architecture

We have developed a metadata storage, retrieval and search system called the Open Science Data Framework (OSDF). OSDF is a specialized system for managing a collection of data files using a Representational State Transfer (REST) Application Programming Interface (API) and JSON, allowing users to easily store, retrieve, query and track changes to data. OSDF is documented at http://osdf.igs.umaryland.edu/docs.php. Apache CouchDB (https://couchdb.apache.org/) is used for the bulk storage of documents, or nodes as they are referred to in OSDF, as well as the storage of each document’s version history.

**Figure 1.**
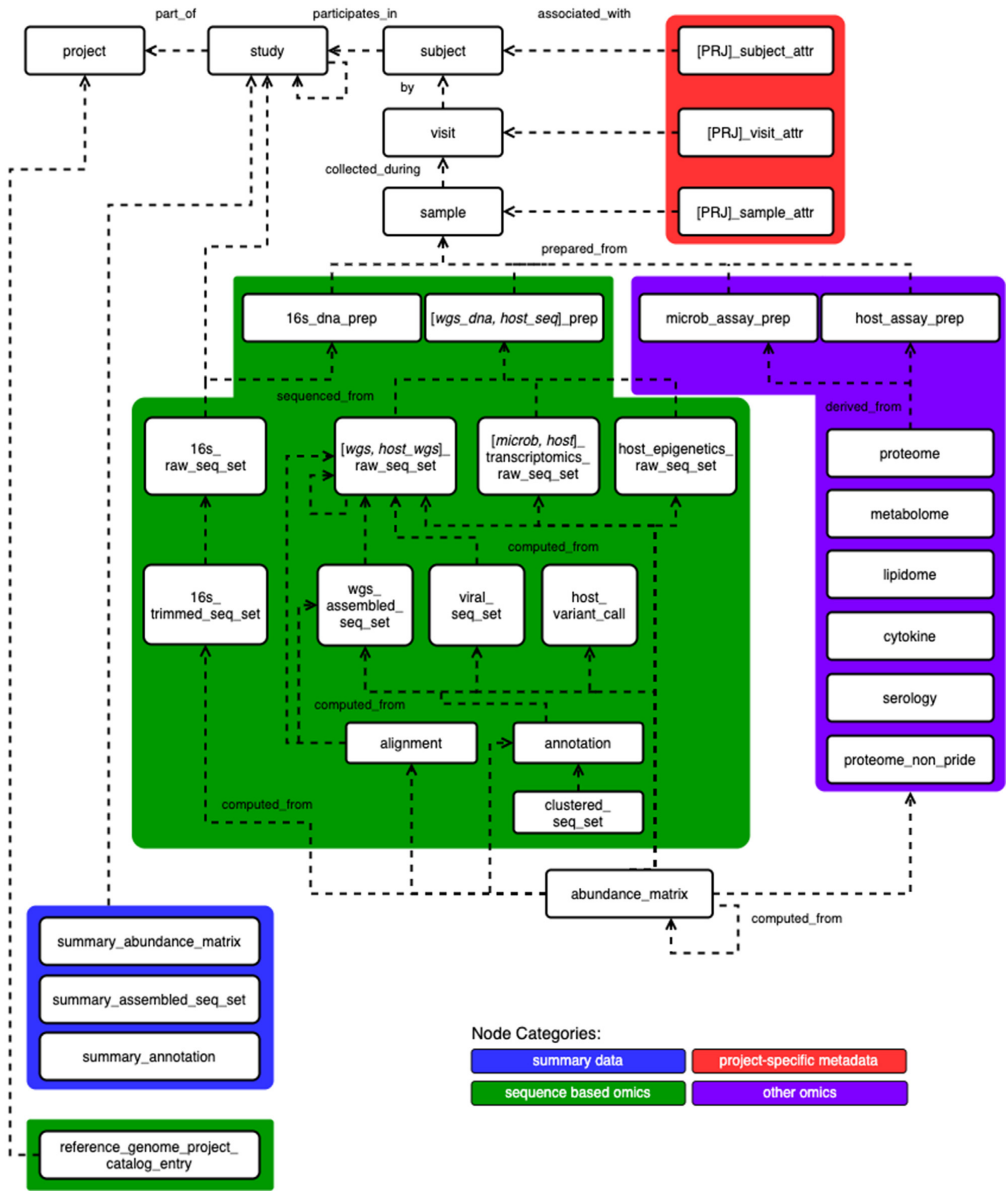
HMPDCC OSDF entity relationship data model. This figure represents the organization of data types listed in Table 1 along with higher level entities. Each white box represents an object, or a node as they are referred to in OSDF, with lines representing the relationships, or linkages, between nodes. Linkages can be traversed as in the following example: [samples] are collected_during a [visit] by a [subject] who participates_in a [study] that is part_of a [project]. Nodes with a red background represent project-specific subject, visit or sample metadata, which became very important during the iHMP when each study involved vastly different phenotypes requiring collection of different types of clinical metadata. Nodes with a green background represent sequence-based omics data types. Nodes with a purple background represent other omics data types. Nodes with a blue background represent summary data aggregated over multiple samples/subjects within a project or study, for example study level BIOM files. Each node depicted here in turn has a corresponding metadata schema that defines required properties, for example subject_id, checksum, or date file path. Schemas are freely available at https://github.com/ihmpdcc/osdf-schemas

The HMPDACC worked closely with data managers from each of the HMP institutions to develop a model and associated schemas to capture data types and metadata associated with relevant objects for the project (e.g. Subject, Sample, RawSequenceSet) (Figure 1). Each box, or node, represents one of these objects, with a corresponding JSON-Schema that imposes structure on the data by defining required properties, data types and formats. This structure allows for validation of completeness and, in some cases, correctness of submitted data. Whenever possible, we have defined controlled vocabularies or used existing ontologies to ensure standardization of metadata values across the three projects. In addition to capturing metadata, OSDF provides control over how nodes connect to one another (linkages) to form a traversable and query-able graph for documenting and storing complex relationships between objects. The end result is that the documents and relationships capture and describe complex data more clearly than is typically possible in a traditional relational database. Schemas are freely available at https://github.com/ihmpdcc/osdf-schemas. OSDF also supports powerful query/search capabilities. It features a custom query language, OSDF Query Language (OQL), that allows for complex, yet still human-readable, queries to be entered via the command line and other interfaces. OQL is documented at http://osdf.igs.umaryland.edu/docs.php. In addition to OQL, OSDF supports the JSON based ElasticSearch Query DSL.

### Versioning and access control

For the HMP project, it was essential to be able to version data to track changes over time. Early in the development of the implementation of OSDF, CouchDB was selected as the main document storage system in part because of its inherent ability to version documents. Because versioning in CouchDB does not survive database optimization via compaction, we created our own versioning system on top of CouchDB. This stores previous versions of documents as an attachment associated with a ‘base’ document that always holds the most recent version of the data. All OSDF nodes contain a ‘ver’ JSON field specifying the version.

OSDF provides several security related features to allow modifications to the underlying data only to those individuals that have been assigned privileges to edit the data. Each namespace has its own access control list configuration that specifies which usernames belong with which control list group. OSDF also allows the system administrator to configure operation of the server with Transport Layer Security encryption so that the transport layer can be secured against monitoring of the network traffic.

## DATA QUERY AND ACCESS

To enable data exploration, searching and access we have built two query tools, the Free Text Search Tool available through the HMPDACC website, and the HMP Data Portal Query Tool.

### The HMPDACC website free text search

**Figure 2.**
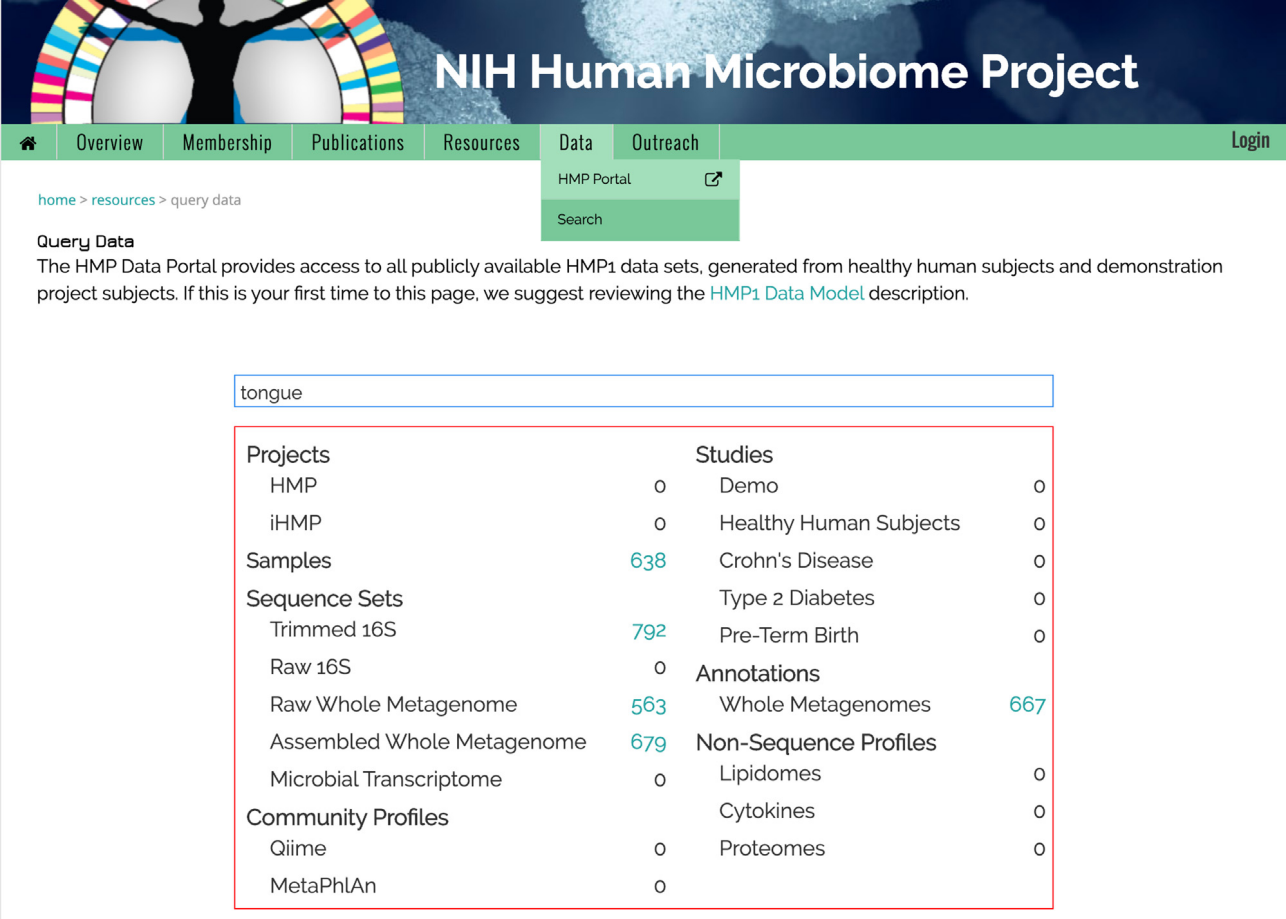
The Free Text Search tool is available under the Data tab of the HMPDACC website. This tool allows for exact or wildcard text searches of metadata fields associated with data elements. The interactive data table is dynamically updated as search terms are entered, allowing users to click and view selected elements.

The free text Search option, available under the Data tab of the HMPDACC website, allows users to search for data based on text found in any of the metadata fields associated with a data element. The free text search is enabled through Elasticsearch (https://github.com/elastic/elasticsearch), a technology that builds indices by scanning text documents and enabling full-text searches. We have deployed an instance of Elasticsearch to index all metadata associated with the data stored in OSDF. This allows us to use a simple text-based search engine to find all data elements matching the query text. The search can be based on exact text search or wildcard text search. As a user types the text in the search box, an associated data summary table is updated to show the number of data elements that match the search text. See Figure 2 for an example search for data associated with the term ‘tongue’. The search result table is dynamically updated to show the number of available elements, in this case associated with sample, raw, assembled and annotated metagenomic wgs sequence sets. A user can click within the table to see a list of particular data elements. The results page is context dependent, with downloadable data sets displayed as a table, and descriptive objects such as projects or studies displayed as short, expandable summaries.

### The HMP Data Portal faceted search and advanced query tools

**Figure 3.**
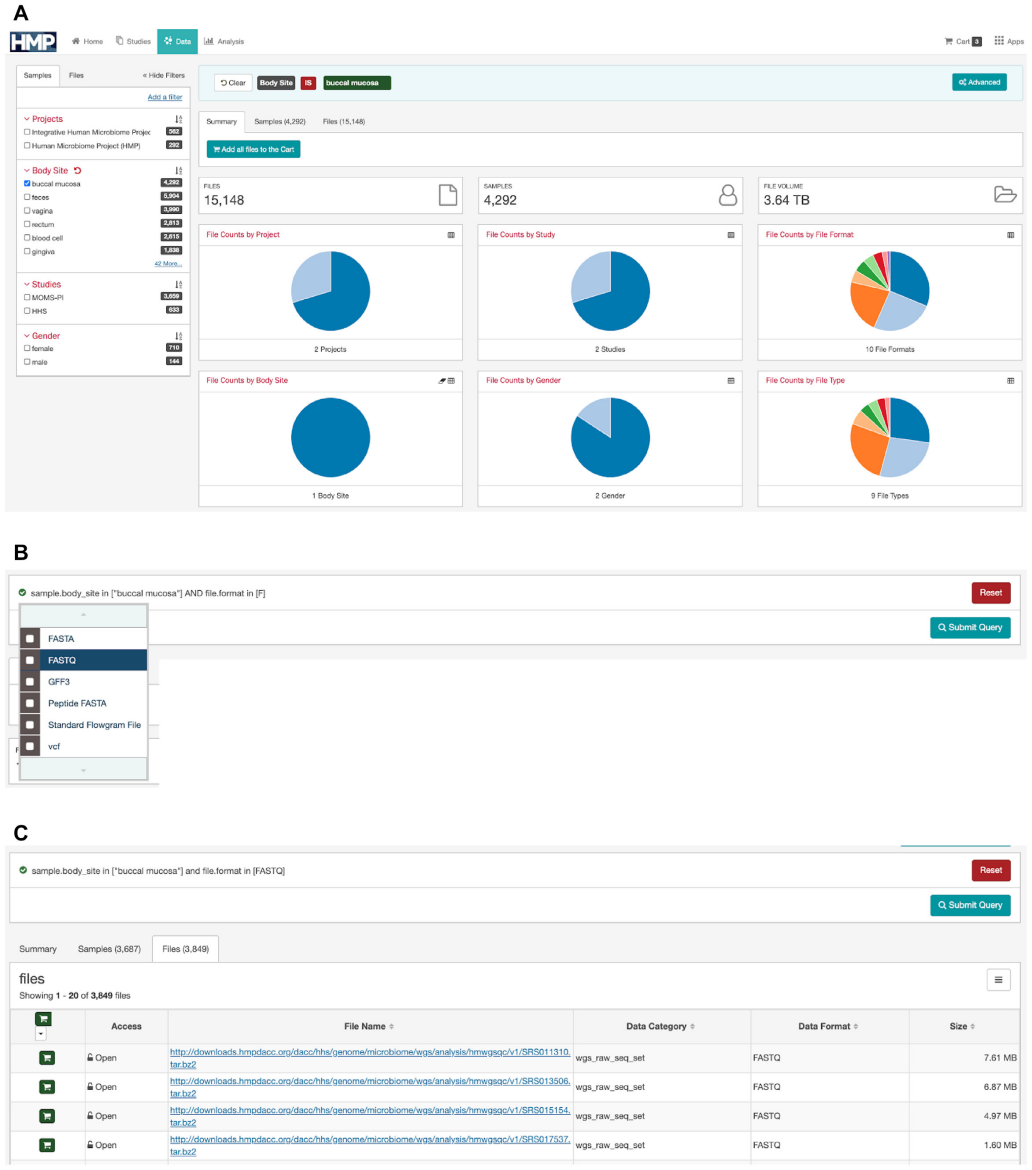
Identifying a dataset of interest, step by step, using the HMP Data Portal Faceted Search and Advanced Query Tools. (**A**) The simple faceted search allows for point-and-click query building with dynamic, interactive graphical summaries of query results; (**B**) advanced search features allow for more customized queries; (**C**) files added to the data cart can be downloaded individually or via a manifest file.

The HMP Data Portal faceted search and advanced query tools enable users to explore data at the HMPDACC in a more customized way. As a starting point for the HMP Data Portal, we used the code-base for the Genomics Data Commons (GDC) Data Portal developed by the National Cancer Institute (https://portal.gdc.cancer.gov/, https://github.com/NCIP/gdc-docs/blob/develop/README.md). We developed our portal by customizing the initial code to meet HMP data needs. To help users find data, the HMP portal uses a structured query approach. The interface offers a simple faceted search mode as well as an advanced search mode. The faceted search uses a predefined subset of curated popular metadata fields as facets, including study, body site, gender, file format and analysis type. An example of the faceted search is shown in Figure 3A. Through a simple point-and-click interface, the user can get a summary of the number of samples, file types and data sizes matching their selected criteria. The summary is displayed graphically as a set of interactive pie charts that the user can click on to further refine the search. Once a user has identified a set of files of interest, the user can add this set to a data cart for further operations.

For power users, or users wanting a more granular search, the site also allows an advanced search feature where all metadata fields tracked in the database are available for searching. The advanced search, accessible by the button at the right of the faceted query box, has an intuitive type-ahead feature such that when the user starts typing text, the interface shows all the fields that match the text string. In addition to this feature, we maintain a list of all the searchable metadata fields on the portal. An example of this is shown in Figure 3B where the search box shows that we are narrowing down our search for the body site ‘buccal mucosa’ to only fastq files. The list of valid file formats are shown in the dropdown box where a user can choose one or more of the file formats. When the user completes the query, the query engine automatically validates the query and flags syntax errors. When the user is ready, they can submit the query and immediately view the updated results summarized by pie-charts. The user can then add the search results to the data cart (Figure 3C). The HMP Data Portal's data cart can be used to accumulate search results for downstream operations. Files in the data cart can be downloaded individually, or, for a collection of files, via download of a manifest file that can be used by a command-line client (https://github.com/IGS/portal_client) to perform bulk downloads to the user’s local system, a cloud environment, or for export to downstream analytical tools. The command-line client does have a 10 000 file download limit. For users wishing to download larger datasets, raw or processed HMP data are accessible, respectively, from SRA or Amazon Web Services (AWS) at s3://hmpdcc/, for download using AWS native Command Line Interface tools.

## FUTURE DIRECTIONS AND CONCLUSIONS

### Common Fund Data Ecosystem

In addition to the HMP, the NIH Common Fund has supported numerous other programs that also generate large quantities of data and have associated Data Coordination Centers (DCCs) (e.g. GTEx (21), LINCS (22)). A new Common Fund project, the Common Fund Data Ecosystem (CFDE) (https://commonfund.nih.gov/dataecosystem), has been developed to provide an overarching cloud-based data infrastructure and framework that will support past, present and future Common Fund project DCCs. The CFDE, in association with the NIH STRIDES program (https://datascience.nih.gov/strides), is developing a cloud-based platform where DCCs can store, and users can access and compute on, Common Fund DCC metadata. Part of this effort is the development of a cross-cutting metadata model (C2M2) that will store metadata associated with all DCC assets. For DCCs that have reached the end of their funded time, not only metadata, but also primary and derived data, will be housed by the CFDE. Some of these data may be controlled-access. A CFDE data portal is under development that will provide controlled access, via portal query and API, to both public and protected data. This will be managed through a system that authenticates users based on whether they have been granted access permissions by the relevant NIH Data Access Committees.

Through these efforts, CFDE will maximize the impact of the resources produced by Common Fund projects and ensure their continued FAIRness (Findability, Accessibility, Interoperability and Reusability) into the future. Organization and management of these efforts is being carried out by the Common Fund Data Ecosystem (CFDE) Coordinating Center (CFDE-CC, O.W. serves as Principal Investigator). In our roles as both the DCC for the Common Fund Human Microbiome Project (HMP) and as part of the CFDE-CC, we are using HMP data in building and testing the CFDE resource. The activities associated with this work include, but are not limited to: (i) using HMP data to evaluate the C2M2 model’s ability to properly represent DCC data, including both publicly available and protected-access data; (ii) loading of both HMP publicly available and protected-access data into the C2M2 and testing the ability of the CFDE query portal to properly control access to subsets of the HMP data by users with different access permissions; (iii) testing the ability of the CFDE query portal to correctly direct users where to find, and how to access, HMP data; and (iv) engaging in harmonization of metadata across CFDE DCCs both for the further development of the C2M2 and for improving FAIRness of Common Fund data. The eventual full integration of HMP data into the CFDE will ensure the continued availability and maintenance of the data into the future. Our activities within CFDE also extend to process documentation and sharing of lessons learned. Coordinating data generated by multiple institutions, over two major projects and nine years leads to a lot of ‘if we knew then what we know now’ moments. We hope to be able to help future DCCs avoid some of the issues that we’ve encountered.

### Reprocessing of phase 1 HMP sequence data

A significant portion of HMP analysis data was generated with older tools that are no longer considered current state-of-the-art. Therefore, as part of our work under the CFDE, we will be re-processing all 16S and whole metagenome sequencing data from the first phase of the HMP using new pipelines incorporating state-of-the-art tools, including those described above in the Tools and Protocols Section. New analysis results will be made available through our HMPDACC resource and eventually the CFDE.

## CONCLUSION

All data on the HMPDACC is available for unrestricted use. The DCC efforts are consistent with NIH’s stated goals to make data generated from NIH funding Findable, Accessible, Interoperable and Reusable (FAIR). The success of these efforts is evidenced by a consistently high rate of user-access to the web resources with around 120 000 user sessions each year. The DCC web resource has a feedback form which users can employ to ask questions or make suggestions for the system. There is a steady stream of user questions coming into the site, with ∼75 received over the past year (as of 8/20). In addition, our institute regularly offers multiple hands-on training workshops focused on various omics and bioinformatics applications. A workshop focused specifically on microbiome analysis is held annually with ∼25 researchers attending each year. The HMPDACC data, protocols, query tools, and associated training opportunities together make up a unique resource for researchers interested in carrying out human microbiome research.

## DATA AVAILABILITY

HMPDACC resources are freely available at www.hmpdacc.org.

## Notes

Present address: James Matsumura, Dina, Chicago, IL 60602, USA.
